# Free Fatty Acids Induce Endoplasmic Reticulum Stress-Mediated Apoptosis of Macrophages in Dairy Cows with Ketosis

**DOI:** 10.3390/ani16071070

**Published:** 2026-04-01

**Authors:** Hongdou Jia, Xinyuan Sun, Wantong Cheng, Yue Yu, Yutong Wu, Jiayi Yan, Yunhui Fan, Qiushi Xu, Juan J. Loor, Chuang Xu, Shixin Fu, Xudong Sun

**Affiliations:** 1 Heilongjiang Provincial Key Laboratory of Prevention and Control of Bovine Diseases, College of Animal Science and Veterinary Medicine, Heilongjiang Bayi Agricultural University, No. 5 Xinfeng Road, Daqing 163319, China; 2 Mammalian NutriPhysioGenomics, Department of Animal Sciences, Division of Nutritional Sciences, University of Illinois, Urbana, IL 61801, USA; 3 College of Veterinary Medicine, China Agricultural University, Beijing 100193, China

**Keywords:** dairy cows, ketosis, macrophage, endoplasmic reticulum stress

## Abstract

Dairy cows with ketosis display immune dysfunction and a high incidence of infectious diseases, which may partly be attributed to excessive endoplasmic reticulum stress (ERS) and apoptosis in macrophages. The objective of the present study was to assess the role of ERS in macrophage apoptosis of ketotic dairy cows. Compared with healthy cows, the apoptosis number of macrophages and the protein abundance of glucose regulated protein 78 (GRP78), activating transcription factor 4 (ATF4), and activating transcription factor 6 (ATF6); the ratio of phosphorylated protein kinase RNA-like endoplasmic reticulum kinase (p-PERK)/PERK, phosphorylated inositol-requiring enzyme 1 (p-IRE1)/IRE1 and phosphorylated eukaryotic translation initiation factor 2α (p-eIF2α)/eIF2α; and mean fluorescence intensity of C/EBP homology protein (CHOP) were greater in cows with clinically ketosis (CK). Treatment with FFA increased protein abundance of GRP78, CHOP, ATF6 and p-IRE1/IRE1, and mean fluorescence intensity of CHOP. Furthermore, FFA increased the protein abundance of cysteinyl aspartate-specific proteinase-3 (Caspase-3) and mean fluorescence intensity of Caspase-3 but decreased the Bcl-2/Bax protein abundance ratio, which was accompanied by an increase in the number macrophage apoptosis. Inhibition of ERS via TUDCA attenuated the increased macrophage apoptosis and the activated apoptotic pathways induced by Tn or FFA. Thus, hyperphysiological concentrations of FFA induce apoptosis in macrophages by triggering ERS in ketotic dairy cows.

## Simple Summary

Ketosis severely impairs the immune function of dairy cows during the periparturient period, increasing their risk of infections. A key driver is the excessive release of free fatty acids (FFA), which are linked to immune cell death. This study explored whether endoplasmic reticulum stress (ERS) mediated FFA-induced apoptosis in bovine macrophages and whether blocking ERS could reverse this damage. Using primary macrophages from ketotic dairy cows and the BoMac cell line, we confirmed that high FFA levels strongly activated ERS pathways (GRP78, CHOP, ATF6, p-IRE1) and triggered apoptosis via the Caspase-3 pathway. Importantly, treating cells with tauroursodeoxycholate (TUDCA), an ERS inhibitor, effectively suppressed these stress responses and reduced macrophage apoptosis. These results highlight ERS as a critical therapeutic target for mitigating immune dysfunction in ketotic dairy cows, offering a potential strategy to enhance herd health and disease resistance during this critical transition period.

## 1. Introduction

Ketosis is a common nutritional disorder in dairy cows that typically occurs during early lactation [1]. The state of negative energy balance (NEB) in peripartal dairy cows results in body fat mobilization [2] and leads to hyperphysiological concentrations of free fatty acids (FFA) that often lead to ketosis [3]. As a result, dysfunction of the immune system can occur and increase the susceptibility of the cow to infectious diseases [4]. Indeed, investigations have revealed that the risk of mastitis in ketotic dairy cows with hyperlipidemia is one–three times greater [5,6].

Macrophages are important functional cells in the innate immune system, which are associated with numerous physiological responses [7,8]. The stability of macrophage numbers is important for proper immune function including initiation of an immune response and aiding in tissue repair and remodeling [9]. Excessive apoptosis results in a decrease in the macrophage numbers, which leads to immune dysfunction and high incidence of infectious diseases. Severe metabolic stress can initiate the process of cell apoptosis [10], and in nonruminants a high level of FFA due to metabolic stress induced apoptosis of macrophages [11]. In one of our previous studies, we detected increased apoptosis of monocytes/macrophages in ketotic cows with hyperlipidemia [12]. Thus, metabolic stress may be an important factor leading to macrophage apoptosis and contributing to a high incidence of infectious diseases.

Endoplasmic reticulum stress (ERS) is a conserved cellular stress response triggered by impaired endoplasmic reticulum (ER) homeostasis, which is mainly featured by the accumulation of misfolded or unfolded proteins, disrupted calcium homeostasis and dysregulated lipid metabolism in the ER lumen [13]. To alleviate ER dysfunction and re-establish cellular homeostasis, ERS initiates the unfolded protein response (UPR), an evolutionarily conserved adaptive signaling network in eukaryotes [14]. The UPR is mediated by three classic ER-resident transmembrane sensor proteins, including activating transcription factor 6 (ATF6), protein kinase RNA-like ER kinase (PERK) and inositol-requiring enzyme 1 (IRE1); these sensors drive distinct yet interconnected signaling cascades to regulate gene expression, protein folding potential and cellular fate [15,16]. High-yielding dairy cows with NEB develop ERS, leading to impairment and dysfunction of metabolically active organs [17]. Elevated circulating FFA levels promote cellular ERS, exacerbate lipid accumulation in bovine hepatocytes and thereby increase the risk of fatty liver disease [18]. Additionally, the mammary gland and mammary epithelial cells of dairy cows are subjected to ERS challenges induced by ketosis and elevated FFA concentrations, contributing to apoptosis and reduced milk yield [19]. Long-term or severe ERS promote these signaling pathways to induce proapoptotic signals [20]. When there is severe ERS, the protein IRE1α separates from glucose-regulated protein 78 (GRP78) and undergoes oligomerization and autophosphorylation [21], which then activates regulated kinase 1 and c-Jun N-terminal kinase [22]. In addition, activated IRE1α cleaves XBP-1 mRNA, promotes its maturation, and induces CHOP expression, all of which cause apoptosis [23,24]. In addition to these mechanisms, Caspase-12 is activated by the CHOP-ERO1α pathway, which further cleaves Caspase-9 and activates a series of caspase effector proteins, also promoting apoptosis [25].

In nonruminants, activation of ERS by high dietary levels of copper sulfate results in hepatocyte apoptosis via triggering the CHOP/Caspase apoptotic pathway [26], while isosteviol (a natural diterpenoid) prevents FFA-induced apoptosis by inhibiting ERS [27]. In this context, work with ruminants has revealed that 5-aminolevulinic acid (5-ALA), endogenous non-proteinogenic amino acid, protects bovine mammary epithelial cells against apoptosis by alleviating ERS [28]. Together, the data suggest that controlling the onset of ERS is key for the prevention of apoptosis in ruminant and nonruminant cells.

Our general hypothesis was that high levels of FFA induce ERS, which may trigger macrophage apoptosis in ketotic dairy cows. The aim of this study was to investigate (1) the status of ERS and apoptosis in monocyte-derived macrophages from ketotic dairy cows and (2) the role of ERS in FFA-induced macrophage apoptosis at the cellular level in vitro.

## 2. Materials and Methods

### 2.1. Animals and Sampling

The Holstein cows used in the experiment were selected as natural cases from a private commercial dairy farm (Heilongjiang Mudanjiang Agricultural Reclamation Jin’ao Dairy Cow Breeding Professional Cooperative, Mudanjiang, China), with similar days in lactation (median = 8 d, range = 3–14) and parity (median = 3, range = 2–4), consistent with the sampling period for dairy cows in previous studies [29,30,31]. On-farm veterinarians screened a total of 99 dairy cows during the sampling period via clinical symptoms and ketone powder method, which were divided into two groups as follows: 52 cows suspected of clinical ketosis (CK), with ketone powder method-positive, accompanied by clinical manifestations of depressed appetite and reduced milk yield, and no concurrent comorbidities; and 47 clinically healthy cows with ketone powder method-negative, without any detectable comorbidities or abnormal clinical manifestations. Based on previous studies [29,30,31] demonstrating statistically significant differences in production and molecular analyses using this cohort selection strategy, 10 clinically ketotic cows with the diagnostic criteria including serum β-hydroxybutyrate (BHB) concentration > 3.0 mM, serum FFA concentration > 0.5 mM, serum glucose (GLU) concentration < 3.5 mM, and 10 healthy cows, with the screening criteria including serum BHB concentration < 0.6 mM, serum FFA concentration < 0.4 mM, GLU concentration > 3.5 mM, were further selected from the two suspected groups.

Blood samples were collected from the coccygeal vein via venipuncture before morning feeding for 3 consecutive days: prior to collection, the skin at the sampling site was cleaned, disinfected with povidone-iodine, and subsequently deiodinated with alcohol; samples were then drawn into evacuated 10 mL anticoagulant-free tubes using 20-gauge Vacutainer needles (Becton Dickinson, Franklin Lakes, NJ, USA), and clean balls were applied to the puncture site for hemostasis after collection. The collected blood samples were centrifuged at 3500× *g* for 15 min at 4 °C.

The concentrations of glucose, BHB and FFA in serum were detected with a Hitachi 7170 automatic analyzer (Hitachi, Tokyo, Japan) with commercially available kits (glucose, GL3815; BHB, RB1008; FFA, FA115; Randox Laboratories, Crumlin, UK). The daily milk yield was recorded by an automatic milking system, and the DMI was recorded daily. Information about experimental cows is reported in Table 1.

### 2.2. Isolation of Blood Mononuclear Cells and Differentiation into Macrophages

In experiment 1, to assess the apoptosis and ER status of macrophages in dairy cows with CK, the monocytes were isolated from blood and subsequently differentiated into macrophages as reported previously by our group [32]. In brief, blood samples were collected by tail vein puncture into a tube containing heparin sodium, and monocytes were isolated from the collected blood samples using a bovine peripheral blood monocyte isolation kit (P5280, Solarbio, Beijing, China). The sterile centrifuge tubes (50 mL) containing blood samples, solution A and solution D (A:D = 3:2) were centrifuged at 800× *g* for 30 min. Subsequently, erythrocytes were lysed with red blood cell lysate (R1010, Solarbio, Beijing, China). The bovine anti-CD14 monoclonal antibody (MCA2678GA, Bio-Rad, Hercules, CA, USA) was used to incubate the cells at 4 °C for 30 min, followed by centrifugating at 250× *g* for 5 min. Next, cells were incubated with MicroBeads (130-407-101, Miltenyi Biotec, Bergisch Gladbach, Germany) at 4 °C for 15 min. The phosphate-buffered solution (PBS) supplemented with 2.0 mM ethylene diamine tetraacetic acid (EDTA) and 5 g/L bovine serum albumin (BSA) was used to resuspend the cells, followed by isolating cells with MACS MS columns (Miltenyi Biotec, Bergisch Gladbach, Germany). The isolated CD14^+^ monocytes were cultured in RPMI-1640 medium (RNBJ7889, Sigma-Aldrich, St. Louis, MO, USA) containing growth-promoting molecules (0.05% α-lactose, 0.05% whey protein and 1 mg/L progesterone), 10% fetal bovine serum (FBS, FB15015, Clark Bioscience, Richmond, VA, USA), 20 ng/mL of bovine granulocyte-macrophage colony-stimulating factor (ab209168, Abcam, Cambridge, MA, USA), 10 μg/L insulin RNBJ7889 (Sigma-Aldrich, St. Louis, MO, USA), 1% penicillin (P1400, Solarbio, Beijing, China) and 20 ng/mL bovine GM-CSF (ab209168, Abcam, Cambridge, MA, USA) for 7 d to differentiate into macrophages.

### 2.3. Cell Culture and Treatments

In experiment 2, to examine the effects of FFA on ERS and apoptosis of macrophages, exogenous FFA were used to treat cells to simulate the state of hematology of cows with CK. The FFA mixture containing stearic acid (7.6 mM, S4751, Sigma-Aldrich, St. Louis, MO, USA), palmitoleic acid (2.8 mM, P9417, Sigma-Aldrich, St. Louis, MO, USA), oleic acid (22.9 mM, O1383, Sigma-Aldrich, St. Louis, MO, USA), linoleic acid (2.6 mM, L1012, Sigma-Aldrich) and palmitic acid (16.8 mM, P5585, Sigma-Aldrich, St. Louis, MO, USA) was dissolved in KOH (0.1 mM), followed by adjusting the pH to 7.4 by hydrochloric acid, as previously reported [33]. To obtain the BSA–FFA complexes, the heated FFA mixture was added into RPMI-1640 medium supplemented with 2% BSA. The BoMac cells (bovine macrophage cell line) were treated with 0, 0.6, 1.2 or 2.4 mM FFA for 12 h, the dose of FFA was chosen according to our previous study [32].

In experiment 3, to investigate the role of ERS in macrophage apoptosis, BoMac cells were treated with the ERS activator tunicamycin (Tn, HY-A0098, MCE, Monmouth Junction, NJ, USA) and the ERS inhibitor tauroursodeoxycholate (TUDCA, HY-19696, MCE, Monmouth Junction, NJ, USA). Briefly, cells were cultured with 500 μg/mL TUDCA or DMSO (solvent, control) for 12 h followed by treatment treated with 2 μg/mL Tn for 12 h.

In experiment 4, to investigate the role of ERS in macrophage apoptosis induced by FFA, TUDCA was used to treat BoMac cells. Briefly, BoMac cells were cultured with 500 μg/mL TUDCA or DMSO (solvent, control) for 12 h followed by treatment with 1.2 mM FFA for 12 h.

### 2.4. Immunofluorescence Staining

The immunofluorescence staining was done as reported in one of our previous papers [34]. In brief, after fixing with paraformaldehyde (4%) for 20 min, cells were incubated with 500 μL Triton X-100 (T9284; Sigma-Aldrich, St. Louis, MO, USA) for 10 min. Cells were then incubated with primary antibodies for CHOP (15204-1-AP, Proteintech, 1:50) and Caspase-3 (ab184787, Abcam, Cambridge, MA, USA, 1:50) overnight, followed by incubating with goat anti-rabbit IgG conjugated with Cy3 dye (A0516, Beyotime Institute of Biotechnology, Shanghai, China, 1:200) for 30 min. BoMac cells were then incubated with Hoechst 33342, followed by capturing fluorescence with laser confocal microscopy (TCS SP8, Lei Ka, Wetzlar, Germany).

### 2.5. Protein Extraction and Western Blotting

The RIPA (P0013B, Beyotime Biotechnology, Shanghai, China) and PMSF (ST506, Beyotime Biotechnology, Shanghai, China) were used to isolate total protein from the cells at a ratio of 99:1. The concentration of protein was detected using a BCA protein concentration determination kit (P0010S, Beyotime Biotechnology, Shanghai, China). Subsequently, an equivalent amount of protein sample (30 μg per lane) was separated with SDS-PAGE, and the gel was transferred to a PVDF membrane (0.45 μm, IPVH00010, Millipore, Burlington, MA, USA). The membranes were sealed in skim milk on a shaker for 2 h, followed by incubating with primary antibodies for IRE1 (1:1000; 3294S, Cell Signaling Technology, Danvers, MA, USA), ATF6 antibody (1:1000; ab122897, Abcam, Cambridge, MA, USA), GRP78 (1:2000; 11587-1-AP, Proteintech, Wuhan, China), p-IRE1 (1:1000; 3294, Cell Signaling Technology, Danvers, MA, USA), PERK (1:1000; 3192, Cell Signaling Technology, Danvers, MA, USA), p-PERK (1:1000; 3179, Cell Signaling Technology, Danvers, MA, USA), phosphorylated eukaryotic translation initiation factor 2α (p-eIF2α, 1:1000; 1138, Cell Signaling Technology, Danvers, MA, USA), eIF2α (1:1000; 5324, Cell Signaling Technology, Danvers, MA, USA), activating transcription factor 4 (ATF4) (1:1000; 1073, Cell Signaling Technology, Danvers, MA, USA), Bcl-2 (1:5000; 68103-1-Ig, Proteintech, Wuhan, China), CHOP (1:1000; 15204-1-AP, Proteintech, Wuhan, China), Bcl-2 associated X (Bax, 1:2000; 50599-2-Ig, Proteintech, Wuhan, China), Caspase-3 (1:2000; ab184787, Abcam, Cambridge, MA, USA) and β-actin (1:1000; ab7817, Abcam, Cambridge, MA, USA) at 4°C for 12 h. The membranes were incubated with the anti-rabbit antibody (1:5000, SA00001-2, Proteintech, Wuhan, China) or anti-mouse (1:5000, SA00001-1, Proteintech, Wuhan, China) for 1 h. Ultrasensitive ECL luminescence reagent (WBKLS0100, Millipore, Burlington, MA, USA) and ImageJ 1.54r gel analysis software (National Institutes of Health, Bethesda, MD, USA) were used to observe and quantify the bands.

### 2.6. Cell Viability Assay

Macrophage viability was assessed using a CCK-8 kit (Solabio, Beijing, China). Cells were seeded into 96-well plates at a density of 1 × 10^4^ cells per well with 100 μL of cell suspension per well, followed by treatment as described in the Cell Culture and Treatment Section. After incubation, CCK-8 reagent was added to each well, and the absorbance at 450 nm was measured using a microplate reader (Thermo Fisher Scientific, Grand Island, NY, USA). Data were normalized to the control group.

### 2.7. Flow Cytometry

Macrophage apoptosis was detected using Annexin V-FITC and PI kits (C1062, Beyotime Biotechnology, Shanghai, China). After digesting and washing with trypsin (without EDTA) and PBS, the macrophages were incubated with 10 μL PI, 200 μL binding buffer and 5 μL Annexin V-FITC for 20 min. Apoptosis was detected via flow cytometry (CytoFLEX, BECKMAN COULTER, Brea, CA, USA).

### 2.8. Statistical Analysis

SPSS software (version 22.0; IBM Corp., Armonk, NY, USA) and Graph Pad Prism 7.0 (GraphPad Software, San Diego, CA, USA) were used to analyze the data. The normality and homogeneity of data were analyzed with the Shapiro–Wilk and Levene tests. The Wilcoxon signed-rank test was used to analyze the non-normally distributed data (presented as the median and interquartile range). One-way ANOVA with subsequent Bonferroni correction (4 groups) and independent sample *t*-test (2 groups) were used to analyze the normally distributed comparisons. Linear and quadratic contrasts were used to evaluate dose-dependent effects. *p* < 0.05 was considered as significant.

## 3. Results

### 3.1. Apoptosis and ERS in Macrophages of Dairy Cows with CK

**Figure 1:**
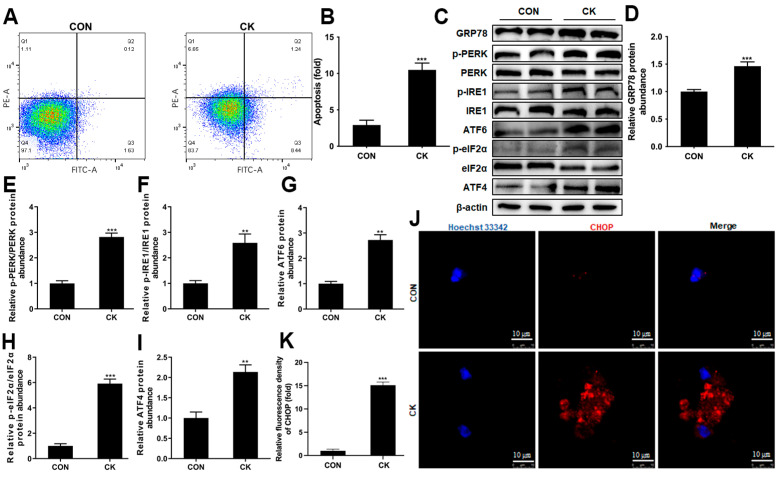
The status of endoplasmic reticulum stress and apoptosis of macrophages of cows with clinical ketosis (CK). (**A**) Flow cytometric analysis of macrophage apoptosis in healthy cows (CON, n = 10) and CK cows (n = 10); (**B**) quantification of macrophage apoptosis (**C**) Western blot analysis of GRP78, p-PERK, PERK, p-IRE1, IRE1, ATF6, p-eIF2α, eIF2α and ATF4; (**D**–**I**) relative protein abundance of GRP78, p-PERK/PERK, p-IRE1/IRE1, ATF6, p-eIF2α/eIF2α, ATF4; (**J**) representative image of immunofluorescence analysis of CHOP (red), Scale = 10 μm; (**K**) quantification of CHOP (red) immunofluorescence. Data were analyzed with an independent sample *t*-test and presented as the means ± SEM. ** *p* < 0.01, and *** *p* < 0.001.

Compared with healthy cows, the apoptosis of monocyte-derived macrophages was greater in cows with CK (*p* < 0.001, Figure 1A,B). The protein abundance of GRP78 (*p* < 0.001, Figure 1C,D), ATF4 (*p* < 0.01, Figure 1C,I), ATF6 (*p* < 0.01, Figure 1C,G) and the ratio of p-PERK/PERK (*p* < 0.001, Figure 1C,E), p-IRE1/IRE1 (*p* < 0.01, Figure 1C,F) and p-eIF2α/eIF2α (*p* < 0.001, Figure 1C,H) were greater in the cows with CK compared with healthy cows. Fluorescence intensity of CHOP was greater in cows with CK compared with healthy cows (*p* < 0.001, Figure 1J,K).

### 3.2. FFA Induced ERS in Bovine Macrophages

**Figure 2:**
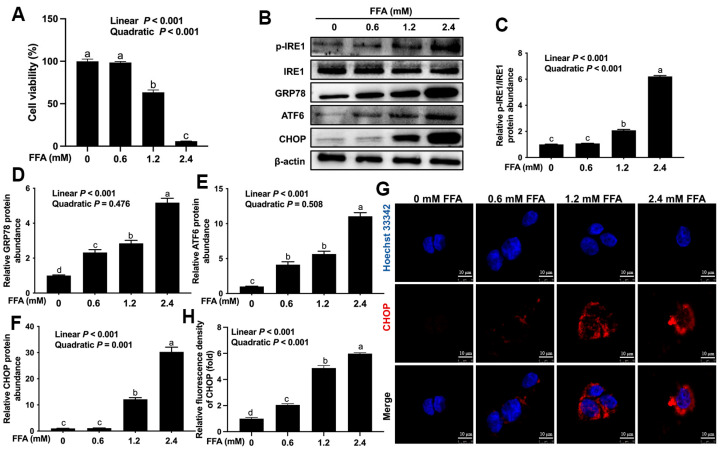
Effects of FFA on endoplasmic reticulum stress of bovine macrophages. BoMac cells were treated with 0, 0.6, 1.2 or 2.4 mM FFA for 12 h. (**A**) Cell viability of BoMac cells; (**B**) Western blot analysis of p-IRE1/IRE1, GRP78, ATF6 and CHOP; (**C**–**F**) relative protein abundance of p-IRE1/IRE1, GRP78, ATF6 and CHOP; (**G**) representative image of immunofluorescence analysis of CHOP (red). Scale bar = 10 μm; (**H**) quantification of CHOP (red) immunofluorescence. Data were analyzed with one-way ANOVA with subsequent Bonferroni correction and presented as the means ± SEM. Different superscript lowercase letters in bar charts indicate a significant difference between groups at the level of *p* < 0.05. Linear and quadratic contrasts were used to analyze dose-dependent effects (*p*-values for linear/quadratic effects are reported in the main text).

Compared with the 0 mM FFA group, the viability of BoMac cells was lower in the 1.2 and 2.4 mM FFA group (Figure 2A; linear, *p* < 0.001; quadratic, *p* < 0.001). Compared with the 0 mM FFA group, the ratio of p-IRE1/IRE1 was higher in the 1.2 and 2.4 mM FFA groups (Figure 2B,C; linear, *p* < 0.001; quadratic, *p* < 0.001). Compared with the 0 mM FFA group, the protein abundance of GRP78 was greater in the 0.6, 1.2 and 2.4 mM FFA groups (Figure 2B,D; linear, *p* < 0.001). Compared with the 0 mM FFA group, the protein abundance of ATF6 was higher in the 0.6, 1.2 and 2.4 mM FFA groups (Figure 2B,E; linear, *p* < 0.001). Compared with 0 mM FFA group, the protein abundance of CHOP was greater in the 1.2 and 2.4 mM FFA groups (Figure 2B,F; linear, *p* < 0.001; quadratic, *p* = 0.001). Compared with the 0 mM FFA group, the mean fluorescence intensity of CHOP was higher in the 0.6, 1.2 and 2.4 mM FFA groups (Figure 2G,H; linear, *p* < 0.001; quadratic, *p* < 0.001).

### 3.3. FFA Induced Apoptosis of Bovine Macrophages

**Figure 3:**
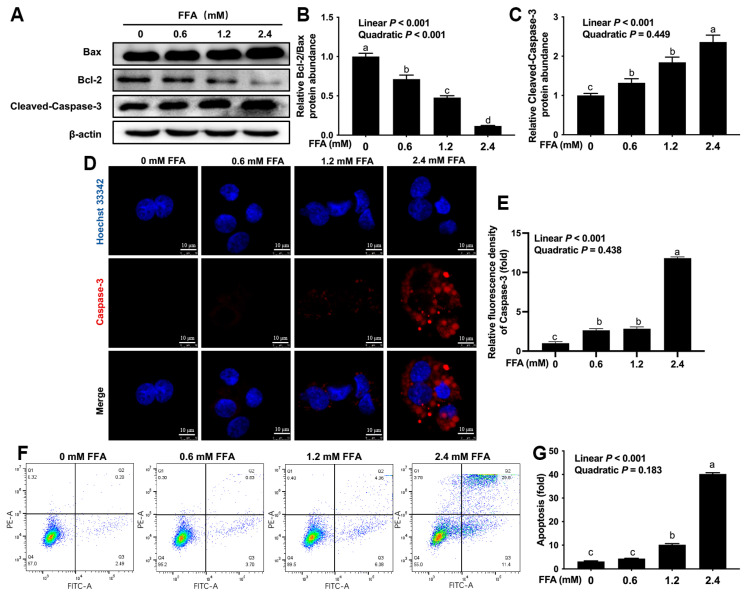
Effects of FFA on apoptosis of bovine macrophages. BoMac cells were treated with 0, 0.6, 1.2 or 2.4 mM FFA for 12 h. (**A**) Western blot analysis of Bcl-2, Bax and cleaved-Caspase-3; (**B**,**C**) relative protein abundance of Bcl-2/Bax and cleaved-Caspase-3; (**D**) representative image of immunofluorescence analysis of Caspase-3 (red). Scale bar = 10 μm; (**E**) quantification of Caspase-3 (red) immunofluorescence; (**F**) flow cytometric analysis of BoMac cells apoptosis; (**G**) quantification of BoMac cells apoptosis. Data were analyzed with one-way ANOVA with subsequent Bonferroni correction and presented as the means ± SEM. Different superscript lowercase letters in bar charts indicate a significant difference between groups at the level of *p* < 0.05. Linear and quadratic contrasts were used to analyze dose-dependent effects (*p*-values for linear/quadratic effects are reported in the main text).

Compared with the 0 mM FFA group, the ratio of Bcl-2/Bax was lower in the 0.6, 1.2 and 2.4 mM FFA groups (Figure 3A,B; linear, *p* < 0.001; quadratic, *p* < 0.001). Compared with the 0 mM FFA group, the protein abundance of cleaved-Caspase-3 was greater in the 0.6, 1.2 and 2.4 mM FFA groups (Figure 3A,C; linear, *p* < 0.001). Compared with the 0 mM FFA group, the mean fluorescence intensity of Caspase-3 was higher in the 0.6, 1.2 and 2.4 mM FFA groups (Figure 3D,E; linear, *p* < 0.001). Compared with the 0 mM FFA group, the apoptosis rate of bovine macrophages was greater in the 1.2 and 2.4 mM FFA groups (Figure 3F,G; linear, *p* < 0.001).

### 3.4. TUDCA Attenuated ERS Induced by Tn in Bovine Macrophages

**Figure 4:**
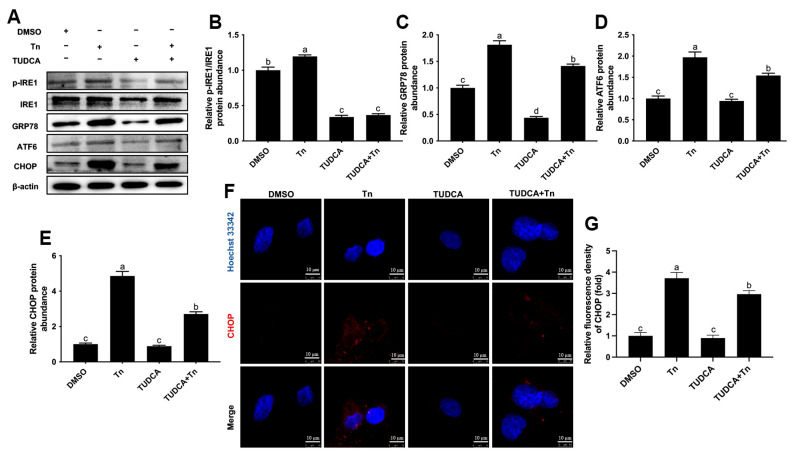
Effects of Tn on endoplasmic reticulum stress in bovine macrophages. BoMac cells were pretreated with 500 μg/mL TUDCA for 12 h and then treated with 2 μg/mL Tn for 12 h. (**A**) Western blot analysis of p-IRE1/IRE1, GRP78, ATF6 and CHOP; (**B**–**E**) relative protein abundance of p-IRE1/IRE1, GRP78, ATF6 and CHOP; (**F**) representative image of immunofluorescence analysis of CHOP (red). Scale bar = 10 μm; (**G**) quantification of CHOP (red) immunofluorescence. Data were analyzed with one-way ANOVA with subsequent Bonferroni correction and presented as the means ± SEM. Different superscript lowercase letters in bar charts represent significant differences (*p* < 0.05).

Treatment with Tn led to a greater protein abundance of p-IRE1/IRE1 (*p* < 0.05, Figure 4A,B), GRP78 (*p* < 0.05, Figure 4A,C), ATF6 (*p* < 0.05, Figure 4A,D) and CHOP (*p* < 0.05, Figure 4A,E), but pretreatment with TUDCA attenuated these responses (*p* < 0.05). Treatment with Tn led to greater fluorescence intensity of CHOP (*p* < 0.05), but pretreatment with TUDCA attenuated this response (*p* < 0.05, Figure 4F,G).

### 3.5. TUDCA Attenuated Apoptosis Induced by Tn in Bovine Macrophages

**Figure 5:**
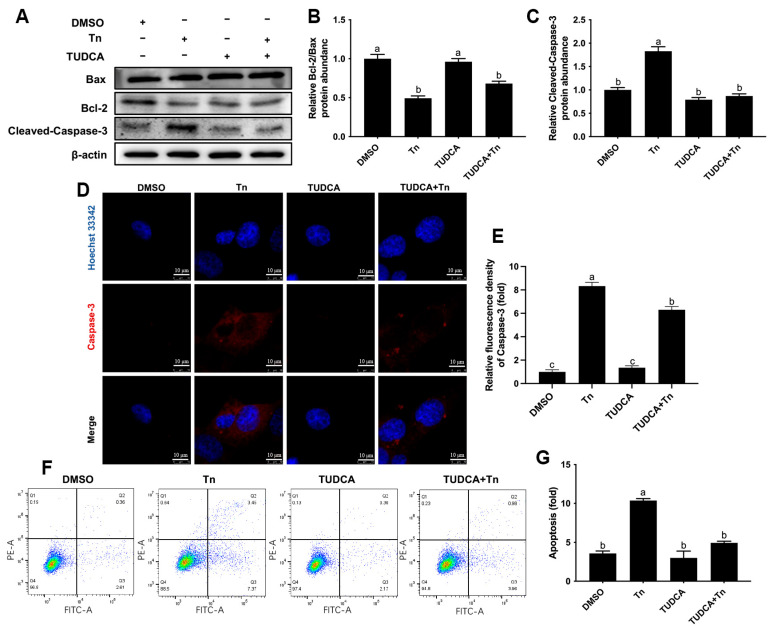
Effects of Tn on apoptosis of bovine macrophages. BoMac cells were pretreated with 500 μg/mL TUDCA for 12 h and then treated with 2 μg/mL Tn for 12 h. (**A**) Western blot analysis of Bcl-2, Bax and cleaved-Caspase-3; (**B**,**C**) relative protein abundance of Bcl-2/Bax and cleaved-Caspase-3; (**D**) representative image of immunofluorescence analysis of Caspase-3 (red). Scale bar = 10 μm; (**E**) quantification of Caspase-3 (red) immunofluorescence; (**F**) flow cytometric analysis of BoMac cells apoptosis; (**G**) quantification of BoMac cell apoptosis. Data were analyzed with one-way ANOVA with subsequent Bonferroni correction and presented as the means ± SEM. Different superscript lowercase letters in bar charts represent significant differences (*p* < 0.05).

Activation of ERS by Tn treatment led to a lower ratio of Bcl-2/Bax (*p* < 0.05) but was attenuated by pretreatment with TUDCA (*p* < 0.05, Figure 5A,B). In addition, treatment with Tn led to greater cleaved-Caspase-3 protein abundance (*p* < 0.05) and of Caspase-3 fluorescence intensity (*p* < 0.05), whereas TUDCA pretreatment attenuated these effects (*p* < 0.05, Figure 5A,C–E). Consistent with the alterations in protein abundance, treatment with Tn led to greater apoptosis of macrophages (*p* < 0.05), but this response was attenuated by the pretreatment with TUDCA (*p* < 0.05, Figure 5F,G).

### 3.6. TUDCA Attenuated ERS Induced by FFA in Bovine Macrophages

**Figure 6:**
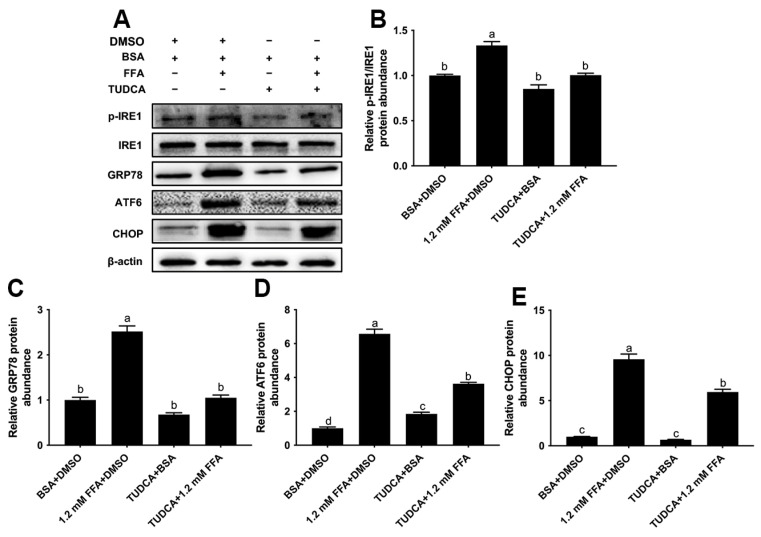
Effect of TUDCA on FFA-induced endoplasmic reticulum stress in bovine macrophages. BoMac cells were pretreated with 500 μg/mL TUDCA for 12 h and then treated with 1.2 mM FFA for 12 h. (**A**) Western blot analysis of p-IRE1/IRE1, GRP78, ATF6 and CHOP; (**B**–**E**) relative protein abundance of p-IRE1/IRE1, GRP78, ATF6 and CHOP. Data were analyzed with one-way ANOVA with subsequent Bonferroni correction and presented as the means ± SEM. Different superscript lowercase letters in bar charts represent significant differences (*p* < 0.05).

Treatment with FFA led to a greater protein abundance of p-IRE1/IRE1 (*p* < 0.05), GRP78 (*p* < 0.05), ATF6 (*p* < 0.05) and CHOP (*p* < 0.05), whereas TUDCA pretreatment attenuated these responses (*p* < 0.05, Figure 6A–E).

### 3.7. Inhibition of ERS Attenuated Apoptosis Induced by FFA in Bovine Macrophages

**Figure 7:**
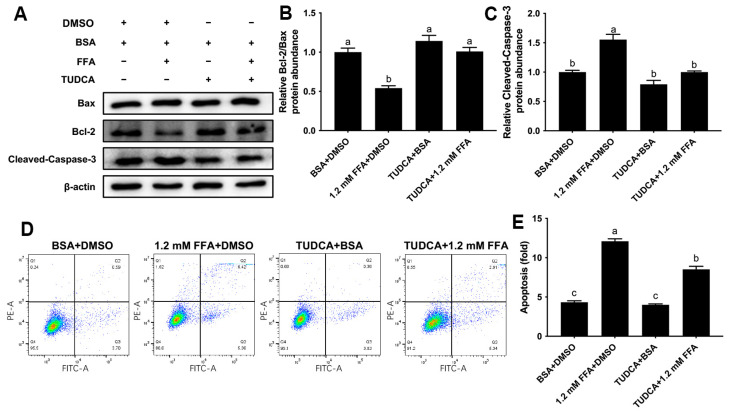
Effect of TUDCA on FFA-induced apoptosis of bovine macrophages; BoMac cells were pretreated with 500 μg/mL TUDCA for 12 h and then treated with 1.2 mM FFA for 12 h; (**A**) Western blot analysis of Bcl-2, Bax and cleaved-Caspase-3; (**B**,**C**) relative protein abundance of Bcl-2/Bax and cleaved-Caspase-3; (**D**) flow cytometric analysis of BoMac cells apoptosis; (**E**) quantification of BoMac cells apoptosis. Data were analyzed with one-way ANOVA with subsequent Bonferroni correction and presented as the means ± SEM. Different superscript lowercase letters in bar charts represent significant differences (*p* < 0.05).

Treatment with FFA led to a lower ratio of Bcl-2/Bax (*p* < 0.05), but this effect was attenuated by the pretreatment with TUDCA (*p* < 0.05, Figure 7A,B). In addition, treatment with FFA led to greater cleaved-Caspase-3 protein abundance (*p* < 0.05), whereas TUDCA pretreatment attenuated these effects (*p* < 0.05, Figure 7A,C). Consistent with the alterations in protein abundance, treatment with FFA led to greater apoptosis of macrophages (*p* < 0.05), a response that was attenuated with the pretreatment with TUDCA (*p* < 0.05, Figure 7D,E).

## 4. Discussion

The metabolic challenges of dairy cows with ketosis during early lactation contribute to an impaired innate immune system, which renders them more susceptible to infections [35,36]. Macrophages are specialized phagocytes with well-established roles in modulation of the host immune system, which represents the defensive forefront line against exogenous and endogenous danger signals [37]. The activity and number of macrophages are central to proper immune function, and in nonruminants metabolic stress-related disease, especially under hyperlipidemia, increases apoptosis of macrophages [38]. The greater apoptosis of macrophages along with the greater circulating concentrations of BHB and FFA in the present study indicated that severe metabolite fluctuations in ketotic cows also are linked to a weakened immune response [35,39] and increased susceptibility of cows to pathogens [40]. Thus, the available data provide a partial explanation for the observed temporal association between an elevated metabolic load during ketosis and their high susceptibility to infectious diseases.

Fatty acids are recognized as potential mediators of immune function [41], with hyperphysiological concentrations of FFA impairing function of peripheral blood mononuclear cells in cows during the transition period, which increased the risk of mastitis [40,42]. At least in nonruminants, high levels of FFA cause apoptosis of macrophages [11,43], which in the present study was also confirmed. Together, the data provide strong support for metabolic stress as a key regulator of immune function in dairy cows [35].

Apoptosis is tightly regulated by a cascade of molecular events [44], with the mitochondrial-dependent apoptotic (Bax/Caspase-3) pathway exerting a considerable impact on this process during metabolic stress [45]. In response to various stimuli, the imbalance of the anti-apoptotic protein (Bcl-2) and pro-apoptotic protein (Bax) drives cytochrome-c release via disruption of the outer mitochondrial membrane permeability [46]. Cytochrome-c, in turn, activates the executioner Caspase-3 that leads to DNA fragmentation and the formation of apoptotic bodies, thereby resulting in cellular apoptosis [47]. In the present study, culturing cells with FFA led to a lower ratio of Bcl2/Bax along with a greater abundance of cleaved-Caspase-3 protein, and Caspase-3 fluorescence intensity was consistent with previous similar findings in both bovine hepatocytes [48] and mammary epithelial cells [33]. Thus, these data highlight that FFA-induced apoptosis of macrophages is partly mediated by activation of the Bax/Caspase-3 pathway.

The endoplasmic reticulum (ER) is a cellular quality-control organelle responsible for maintaining protein homeostasis by regulating protein processing, modification, and folding [49]. GRP78 is a key molecular chaperone localized in the ER lumen, and it plays a central role in orchestrating the ERS response [50]. Under physiological conditions it binds to the three core UPR sensors (PERK, IRE1, and ATF6) to keep them in an inactive state [51]. Disruption of ER protein homeostasis, such as during metabolic stress, leads to the accumulation of misfolded or unfolded proteins which sequester GRP78 and trigger severe ERS [52,53]. This sequestration causes GRP78 to dissociate from PERK, IRE1, and ATF6, thereby initiating UPR signaling cascades. Activated IRE1 undergoes oligomerization and autophosphorylation, ATF6 is translocated to the Golgi apparatus where it undergoes cleavage and subsequent activation, and PERK is activated via autophosphorylation [54]. Collectively, these three ERS-dependent UPR pathways converge to upregulate the expression of CHOP, a key pro-apoptotic protein, and sustained ERS ultimately culminates in cellular apoptosis [55,56]. The greater protein abundance of GRP78, ATF4, ATF6, and the ratio of p-PERK/PERK, p-IRE1 IRE1 and p-eIF2α/eIF2α and fluorescence intensity of CHOP highlighted the existence of ERS in macrophages of ketotic dairy cows, potentially driven by the high levels of FFA during ketosis. This idea is supported by the findings from the present study and previous data reporting increases in phosphorylated PERK and eIF2α and protein abundance of GRP78, ATF4, and CHOP in bovine hepatocytes incubated with FFAs [57,58]. The upregulation of CHOP expression in turn promotes the expression of the pro-apoptotic protein Bax, while inhibiting the expression of the anti-apoptotic protein Bcl-2, which further triggers the Caspase-3-dependent apoptosis events [53]. Taken together, these data lead us to speculate that the increase in macrophage apoptosis in dairy cows with ketosis is associated with FFA-induced ERS.

Severe or persistent ERS represents an important way to evoke cellular apoptosis via activating Bax/Caspase-3 signaling [59], while Tn also can activate ERS within cells [60]. The fact that Tn treatment led to greater protein abundance of GRP78, CHOP and ATF6, and p-IRE1/IRE1 along with greater fluorescence intensity of CHOP in the present study highlights that this molecule also acts as an activator of ERS in bovine macrophages. Work with nonruminants demonstrated that activation of ERS by Tn induced cell apoptosis via the Bax/Caspase-3 pathway [61]. Administration of TUDCA led to a higher ratio of Bcl2/Bax, while it decreased protein abundance upregulation of cleaved-Caspase-3 and apoptosis of macrophages, indicating that ERS directly regulated the apoptosis of bovine macrophages.

Inhibition of ERS is considered a therapeutic approach for mitigating cell apoptosis mediated by metabolic stress [58,62], and TUDCA can inhibit ERS, which restores endoplasmic reticulum function by stabilizing protein structure, promoting protein folding and restoring protein localization [63]. Thus, the lower protein abundance of GRP78, CHOP and ATF6, and p-IRE1/IRE1 suggested that TUDCA was effective in attenuating ERS induced by Tn or FFA. In both nonruminants and ruminants, inhibition of ERS by TUDCA reduced apoptosis by blocking the Bax/Caspase-3 apoptotic signaling pathway [64,65]. In the present study, TUDCA treatment effectively attenuated FFA/Tn-induced downregulation of the Bcl2/Bax ratio and upregulated cleaved-Caspase-3 protein abundance and apoptosis of macrophages, suggesting that FFA induced the Bax/Caspase-3-dependent apoptosis of macrophages via activating ERS. Together, our data support the previous findings that inhibition of ERS plays a cytoprotective role against cellular apoptosis induced by metabolic stress [66].

## 5. Conclusions

Elevation of circulating concentrations of FFA during ketosis causes ERS, which results in apoptosis of macrophages via activation of the Bax/Caspase-3 apoptotic signaling pathway. Thus, ERS constitutes a crucial cause of bovine macrophage apoptosis induced by FFA.

## Figures and Tables

**Table 1:** The basic physiological parameters of the healthy (control, *n* = 10) and clinical ketotic (CK, *n* = 10) cows ^1^.

Item	Control	CK	*p*-Value
Milk yield (kg)	37.6 (3.2)	28.6 (3.7)	<0.001
DMI (kg/d)	23.6 (1.3)	19.8 (1.7)	<0.001
Serum glucose (mM)	4.01 (0.43)	2.33 (0.28)	<0.001
Serum BHB (mM)	0.42 (0.18)	3.35 (0.42)	<0.001
Serum FFA (mM)	0.39 (0.11)	1.09 (0.25)	<0.001

^1^ Data were analyzed by Wilcoxon signed-rank test and presented as the median and interquartile range (IQR, in parentheses).

## Data Availability

The original contributions presented in this study are included in the article. Further inquiries can be directed to the corresponding authors.

## Abbreviations

The following abbreviations are used in this manuscript:

ERS	Endoplasmic reticulum stress
CK	Clinically ketotic
GRP78	Glucose regulated protein 78
CHOP	C/EBP homology protein
FFA	Free fatty acid
ATF6	Activating transcription factor 6
Caspase-3	Cysteinyl aspartate-specific proteinase-3
TUDCA	Tauroursodeoxycholate
Tn	Tunicamycin
NEB	Negative energy balance
UPR	Unfolded protein response
PERK	Protein kinase RNA like ER kinase
IRE1	Inositol requiring protein 1
5-ALA	5-aminolevulinic acid
PBS	Phosphate-buffered solution
EDTA	Ethylene diamine tetraacetic acid
BSA	Bovine serum albumin
FBS	Fetal bovine serum
